# An Agenda for Advancing Research and Prevention at the Nexus of Work Organization, Occupational Stress, and Mental Health and Well-Being

**DOI:** 10.3390/ijerph20116010

**Published:** 2023-05-31

**Authors:** Michael K. Lemke, Adam Hege, Alexander M. Crizzle

**Affiliations:** 1 Department of Social Sciences, University of Houston-Downtown, Houston, TX 77002, USA; 2 Department of Public Health and Exercise Science, Appalachian State University, Boone, NC 28608, USA; hegeba@appstate.edu; 3 School of Public Health, University of Saskatchewan, Saskatoon, SK S7N 2Z4, Canada; alex.crizzle@usask.ca

**Keywords:** work organization, occupational stress, mental health, well-being, research agenda, trucking industry, long-haul truck drivers, Total Worker Health, syndemic theory, complex systems

## Abstract

Work characteristics and worker well-being are inextricably connected. In particular, the characteristics of work organization shape and perpetuate occupational stress, which contributes to worker mental health and well-being outcomes. Consequently, the importance of understanding and addressing connections between work organization, occupational stress, and mental health and well-being—the focus of this Special Issue—increasingly demand attention from those affected by these issues. Thus, focusing on these issues in the long-haul truck driver (LHTD) sector as an illustrative example, the purpose of this commentary is as follows: (1) to outline current research approaches and the extant knowledge base regarding the connections between work organization, occupational stress, and mental health; (2) to provide an overview of current intervention strategies and public policy solutions associated with the current knowledge base to protect and promote worker mental health and well-being; and (3) to propose a two-pronged agenda for advancing research and prevention for workers during the 21st century. It is anticipated that this commentary, and this Special Issue more broadly, will both echo numerous other calls for building knowledge and engaging in this area and motivate further research within complementary current and novel research frameworks.

## 1. Introduction

Work characteristics and worker well-being are inextricably connected. Not only is work considered to be a part of one’s identity [1], it is also considered to be a social determinant of health and health equity [1,2,3], as adults spend a significant amount of their time working and in the workplace [4]. Although one’s work can lead to positive impacts for worker well-being [1,5], it is also known that adverse work conditions greatly contribute to public health challenges [6]. In particular, the characteristics of work organization—defined by the National Institute for Occupational Safety and Health (NIOSH) as “the work processes and the organizational practices that influence job design” [7]—constitute key factors in stress-related exposures and outcomes experienced among working populations [8,9]. Specifically, work organization characteristics shape and perpetuate worker vulnerability to excessive occupational stress—defined as “the harmful physical and emotional responses that occur when the requirements of the job do not match the capabilities, resources, or needs of the worker” [8]—which in turn contributes to poor mental health and well-being outcomes in professions such as long-haul truck driving [6,10].

Consequently, the importance of understanding and addressing connections between work organization, occupational stress, and mental health and well-being—the focus of this Special Issue—increasingly demand attention from occupational safety and health professionals, industry leaders, public health organizations, policymakers, and numerous other stakeholders who are affected by these issues [1]. The gravity of research and preventive solutions in this arena is further reinforced by emerging threats to mental health and well-being among workers. In particular, the COVID-19 pandemic reaffirmed the presence of persistent disparities in these outcomes across worker segments [11,12] and crystallized the critical necessity of evidence-based programming for ensuring the resilience of national and global economies [13,14]. Together, endemic and emerging threats demonstrate the importance of the nexus of work organization, occupational stress, and worker mental health and well-being.

Thus, focusing on these issues in the long-haul truck driver (LHTD) sector as an illustrative example, the purpose of this commentary is as follows: (1) to outline current research approaches and the extant knowledge base regarding the connections between work organization, occupational stress, and mental health; (2) to provide an overview of current intervention strategies and public policy solutions associated with the current knowledge base to protect and promote worker mental health and well-being; and (3) to propose a two-pronged agenda for advancing research and prevention for workers during the 21st century.

## 2. Understanding the Nexus of Work Organization, Occupational Stress, and Worker Mental Health and Well-Being

Globally, nearly 60% of all people are workers [5]. The nature of work, and associated work organization characteristics, has continued to evolve in the US and globally, with major changes to job design occurring in the last several decades [15]. As a result, the incidence and prevalence of mental health issues—which involve changes in emotion, thinking, and behavior (or a combination of these) [16]—have risen, with detrimental impacts on productivity, job satisfaction, relationships, and quality of life [17]. Currently, an estimated 15% of working-age adults have a mental disorder, with depression and anxiety specifically resulting in 12 billion lost working days and 1 trillion USD in lost productivity annually [5]. In the US, workers are working longer work hours, more frequently encountering working schedules that features shift work, enduring greater psychosocial job stressors, and facing increasingly poor work–life balance [18,19]. As a result, in the US, almost 20% of the 160 million older adults aged 18 and older who are employed have a mental health issue [1,20]. These outcomes are disproportionately poor in the US compared to other developed countries and highlight the importance of addressing work organization and its cascading impacts on workers, workers’ families, industry stakeholders, and the US and global economy [1,21]. Studies show that work organization characteristics that include excessive workloads and low job control can lead to feelings of stress, isolation, anxiety, and depression, all of which are risk factors for poor mental health and the development of anxiety and depressive mental health disorders [1,22].

In the past decade, studies specifically show these connections and disparities within the trucking industry. This industry is influenced by neoliberalism, an approach that supports free trade, deregulation, globalization, and a reduction in government spending [23]. Based on neoliberalism, supply chains continue to seek the cheapest forms of labor to maximize profits. This has resulted in workers in this industry—especially long-haul truck drivers (LHTD)—working harder, in worse conditions, and for relatively low wages [23]. Over the past 30 years, LHTD wages have been stagnant, seldom increasing despite rising costs from inflation. Given the current demand for LHTD and the pressing need for them, especially during the COVID-19 pandemic, companies have only recently increased their wages to retain and attract new drivers [24]. At the same time, the advances of technology within the trucking industry have resulted in more automation and regulations. This has made the process of delivering goods more efficient, while also reducing the number of laborers, resulting in less negotiating power of unions to advocate for higher salaries. As a result of these changes over the past several decades, the LHTD profession has become increasingly stressogenic [13].

In the trucking industry, organizational stress resulting from tight delivery schedules, time demands, poor driving conditions, and fatigue, are primary determinants of mental health [6,25,26,27]. These risk factors are further exacerbated by other physical, behavioral, organizational, environmental, and psychosocial environments [28,29]. Additionally, the impact of supply chain pressures (e.g., work demands and scheduling), changing demographics (e.g., more women entering the profession), and regulatory policies can also impact mental well-being [28]. Currently, almost 50% of LHTD report being stressed [10,30], and more than 50% report having poor family relationships [30]. Despite the linkages between the occupational milieu of trucking and mental health, there are limited data on the prevalence of mental health issues in LHTD, although one study did find that 44% of LHTD had depressive symptoms in the past year that were predicted by severe stress, psychiatric medication use, and poor sleep quality [27]. Other studies have similarly reported that LHTD have symptoms of depression, loneliness, and isolation [10,30].

Due to these (and other) forces and worker-level outcomes, the current industry state is impacted by poor productivity, job satisfaction, and recruitment and retention issues in a sector that is experiencing a significant shortage of workers [31,32]. For example, in Canada, it is estimated that there will be a shortage of 55,000 LHTD by the end of 2023 [31]. Similarly, according to the American Trucking Associations, there is a current shortage of 80,000 LHTD in the US [32]. In addition to a retiring workforce, there are significant challenges recruiting new drivers, given issues related to pay and lifestyle. LHTD are also currently leaving the sector to search for jobs with better pay, benefits, and working conditions. Cumulatively, these issues threaten the long-term vitality and resilience of critical supply chains that were already strained during the COVID-19 pandemic [14].

## 3. Interventions and Public Policy Solutions to Improve Worker Mental Health and Well-Being

Over the past several decades, literature from multidisciplinary fields, such as organizational psychology, management, medicine, and public and occupational health disciplines, have explored the intersection of work, occupational stress, and mental health. Much of the research has been informed by two seminal theoretical perspectives: the Karasek model [33] and the Siegrist model [34,35]. The Karasek model takes on a ‘job strain’ approach in which occupation-specific aspects, such as risks (physical, mental, etc.) and the ability to make decisions on the job, are measured and can be categorized into “high strain”, “active job”, “low-strain”, or “passive job” [33]. Occupations with higher demands and stressors coupled with a lower decision latitude are considered to be of the highest job strain; these types of professional characteristics have been associated with the worst health outcomes. Building from this model, Johnson and colleagues [36] brought in the importance of organizational leadership and co-worker social support to add a fifth category of “iso-strain”. This addition suggests that working in an organization culture the provides social or interpersonal support, whether from a supervisor or coworkers, can assist in reducing the impacts of demand and decision latitude imbalance or help a worker cope with the challenges. The Siegrist model focuses on the effort (hours, scheduling, etc.) that workers put into the job in relation to the rewards (pay, promotion, support, etc.) they get in return [37]. This model centers more on the intrinsic nature of individual employees: Some workers are motivated by the effort (flexibility of scheduling, hours conducive to their overall life), whereas others seek increased salary and wages or promotion opportunities. However, organizations can and should pursue a culture that balances the effort and rewards of their workforce. In recent years, prominent theories have also included the Conservation of Resources theory [38], work–life and work–conflict balance [39], and the job demands-resources theory [40,41,42]; in addition, these theories have looked at such aspects as organizational justice, job security factors, job safety, and conflict/bullying in the workplace. Lastly, these theories have also been utilized to measure these psychosocial job stressors in relation to physical and physiological health impacts, such as cardiovascular disease and allostatic load [35].

These theoretical frameworks have been used to inform intervention approaches at the organization level and in public policy. Organizational-level approaches have predominantly focused on secondary and tertiary prevention strategies [34,43]. These range from individual-level focused stress management and coping programs, comprehensive health promotion programs, and screenings to access to mental health care and clinical treatment [43,44]. However, very limited research has documented organizational-level change focused on primary prevention strategies that would include changes such as work design/redesign, work organization, and work management/decision latitude [45,46]. As LaMontagne and colleagues [47] argued, the best approaches are integrative and focus on improved work environment and organization, organizational culture and support, and treatment access—in other words, they are multipronged, informed by multidisciplinary perspectives, and work at multiple levels of intervention. In addition, they can concurrently address both safety and health characteristics of work. Due to organizations primarily emphasizing secondary and tertiary approaches, with limited attention at the primary level, sustainable impacts are rarely found, and studies have largely concluded them to be unsuccessful [48]. Thus, there is an urgent call for concerted efforts aimed at moving from the illness/treatment approach to a health/prevention one [49].

Globally, nations have increasingly recognized the importance of mental health and its linkages with work. In particular, as Schulte and colleagues [50] expressed, as work has continually evolved, that policymakers must understand the linkages between work- and non-work-associated stressors; therefore, the subjective and objective employee characteristics must be considered and that all public policy should consider well-being. Therefore, public policy should emphasize work organization features (e.g., work hours, scheduling practices), occupational safety, work–life balance, payment structures and earnings, social safety nets, and employee participation in decision-making. Further, in their recent review, Blustein et al. [51] suggested a policy landscape that affords everyone the opportunity to not only employment, but also to “decent work and meaningful work” that centers on the whole person. These approaches to public policy-oriented interventions can result in not only an improved societal quality of life, but they can also address systems of oppression and privilege and existing health and social inequities [52].

In recent years, novel and holistic approaches that are shaped by, and inform, public policy and organizational-level changes have emerged. For example, in the US, NIOSH and the Centers for Disease Control and Prevention have adopted the Total Worker Health framework [53], which aims to inform organizational policies and practices and public policy from a broad and comprehensive perspective; in doing so, it seeks to promote healthier and safer workplaces and employees. More recently, and building from the Total Worker Health approach, US researchers associated with NIOSH suggested a more advanced model that centers on overall well-being and seeks to account for the aforementioned work- and non-work-related connections to health and well-being [54]. As such, much more attention is being directed at the social, economic, and political forces in research and policy efforts and how they shape the downstream working conditions and worker health outcomes [28]. However, there is a need for the research to be translated to practice and policy more widely.

Specific to LHTDs, improving working conditions within the trucking industry is critical to retaining and attracting workers. There are various health promotion programs available to LHTD, including the North American Fatigue Management Program [55] and the Certificate of Recognition [56], which address issues related to fatigue and regulatory issues and management systems, respectively; however, neither of these programs focus specifically on mental health. Additionally, prevention efforts have rarely targeted forces that are upstream from driver behavior and education, such as those key work organization characteristics that shape downstream occupational stress and corresponding mental health and well-being outcomes [30,57].

## 4. A Two-Pronged Agenda for Advancing Research and Prevention Efforts

There is still much to be learned and investigated as it relates to understanding how one’s work impacts well-being and mental health and how best to direct prevention efforts. Work is continually evolving to meet the demands of society, and unforeseen societal disruptions can exacerbate underlying mental health challenges of workers [58]. The COVID-19 pandemic in particular has forced us as societies to reevaluate work and the ways that we do it; in addition, it has highlighted underlying disparities found related to work-related health impacts and the linkages with other social determinants of health [59]. For example, shifts in work organization toward remote work during the COVID-19 pandemic were less likely to apply to low-income and racial/ethnic minority workers, who were disproportionately employed in ‘essential’ occupations that were especially vulnerable to work-related stress and negative mental health outcomes [11,60,61,62]. Although the long-term impacts of COVID-19 for work organization, occupational stress, and mental health are continuing to unfold, it is known that changes in work organization during COVID-19 have unintentionally but significantly contributed to the ‘Great Resignation’ [63] by magnifying worker dissatisfaction [64]. More broadly, empirical findings regarding the impacts of the COVID-19 pandemic on worker stress and mental health continue to bring to light how, when a global pandemic or other similar emergent crisis occurs, our approaches must be adaptable. Namely, that we cannot be so reactive, and we must do a better job of proactively preparing for these types of events. One example is preparing for how global climate change can be expected to lead to work-related impacts. For instance, global warming has been associated with reductions in worker productivity worldwide [65], with noted consequences for work capacity and productivity for workers in heat-exposed jobs in particular [66]. Continued climate change can be expected to lead to shifts in work organization as industries adapt, including changes that impact both work practices (e.g., increases in automation) and work opportunities [65,66]. These adaptations may induce negative impacts for workers, such as loss of income, with consequences for work-related stress and mental health and well-being [66]. Meeting this and other emerging challenges will require utilizing data in public and occupational health-related research that may have been overlooked to date, as well as the collecting of new data and engaging in novel research approaches.

Moving forward, to bridge gaps in understanding of the nexus of work organization, occupational stress, and mental health, and to apply this understanding to effective preventive solutions, several basic and applied research initiatives are absolutely vital. In response, we provide several (non-inclusive) future avenues of research and prevention below that constitute potential next steps. These avenues are bifurcated into two paradigmatic “prongs”. Although these two prongs are described below separately, they should be conceptualized as interdependent, complementary, and—ideally—synergistic.

### 4.1. Prong 1: Future Avenues of Research and Prevention within Current Research Frameworks

Future research studies need to be comprehensive and multidimensional to elucidate greater predictive validity. For example, Shahidi and colleagues [67] created latent psychosocial work environment profiles that were able to examine both positive and negative aspects of work and how they interact with each other. This indicates that new and innovative data analytic strategies can play a critical role in understanding the nexus of work organization, occupational stress, and mental health. It is also important to include more non-work-related mental health factors into models (e.g., life satisfaction, community engagement and lifestyle) [54]. Another area of research that needs to be expanded upon comprises the linkages between work stress, mental health, and physical health conditions, and this could also be supported by more intervention studies and longitudinal research [68]. Other emerging challenges to worker mental health include the rapid rise in new forms of technology, economic crises, and new forms of employment and work organization [69]. More broadly, to address mental health challenges, changes are needed that holistically target multiple levels of influence, including the individual, organizational, and societal/public policy levels [70]. This may result in significant action to address mental health challenges and other workforce factors to enhance productivity, efficiency, and job satisfaction across all occupational categories. Specifically, intervention research that evaluates changes in work organization vis-à-vis corporate, regulatory, and other relevant policy change should be prioritized. Although these are often more difficult intervention ‘levers’ to pull, they are also the most likely to generate sustainable population-level change in working conditions compared to individual-focused interventions [71]. Further, when implemented holistically (e.g., in conjunction with individual-level components), work organization-oriented policy interventions may be especially powerful for simultaneously addressing the nexus of work organization, occupational stress, and mental health. Frameworks such as Total Worker Health [72] and syndemic theory [73] (discussed in Section 4.2) can serve as the bases for such novel intervention programming.

There has also been a growing need for workers to have more of a voice in their work environment and working conditions. Participatory approaches, such as participatory action research, can result in improved work conditions and policies and practices and more comprehensive strategies aimed at worker well-being [74]. Incorporating participatory approaches into research and prevention efforts can provide valuable insights into the worker experience and the intersection between work- and non-work-associated stressors, which exacerbate already existing occupational stress. For example, the COVID-19 pandemic added additional stress and trauma to workers, both in the workplace and beyond [58]. There were vast differences in the impacts of the pandemic; for example, some workers worked from home, whereas others continued to work on the frontline to maintain the economy. However, from a global perspective, it is important to note that there are various cultural, political, and economic perspectives that lead to disparities in terms of resources and capacity directed at occupational mental health challenges [75].

Future directions for truck driver research need to address the high morbidity seen in the LHTD sector. Numerous studies show that LTHD have high rates of chronic disease [29,76]. It is likely that poor mental health contributes to the development of chronic diseases in LHTD (e.g., hypertension, diabetes, and cardiovascular disease) [77]; however, the causal factors are unclear as prior studies have typically employed cross-sectional surveys. Large database studies show that psychiatric medication usage in LHTD significantly increases over time [78], suggesting cumulative effects among LHTD that have not been resolved through current programs and health and wellness initiatives.

To date, much of the organizational-focused and public policy approaches in the trucking industry have consistently focused on individual driver health through secondary and tertiary prevention; however, these have been largely ineffective. Although mental health challenges are more recognized, there is still a stigma involved in discussing issues related to mental health in the trucking profession. Trucking is a male-dominated profession, with many males masking their mental health issues for fear of being labeled as weak [79]. Female LHTD also have gender-specific issues, such as discrimination, feelings of being unsafe, and feelings of inadequacy when balancing being a trucker with that of a caregiver [80]. The first steps for improving mental health issues could be to (1) improve the working conditions of LHTD, which are largely shaped by public policy; and (2) to create an organizational and societal culture where talking about mental health challenges is acceptable without judgment. Although largely untapped, efforts toward improving LHTD mental health that are grounded in Total Worker Health approaches may hold promise in bridging the overlapping and potentially exacerbating array of causal forces at play [13]. Participatory approaches could also be valuable for engaging with LHTD in driver-centered research to learn of unique gender-based mental health challenges and would involve drivers meeting with and discussing aspects of their work with both organizational leaders and policymakers or drivers engaging in research collaboratively with a wide range of stakeholders. Incorporating more perspective and voice from working truck drivers would be beneficial in improving policy and allowing for a more flexible and comprehensive approach.

At the organizational level, fleet management could help address mental health issues through the provision of mental health modules and by providing physical and mental healthcare services at distribution centers and truck stops. This would allow trained professionals to meet LHTD where they are. Additionally, the use of telehealth may be beneficial for LHTD who have difficulty attending in-person sessions due to their scheduling demands. This also could include having supervisors and dispatchers check-in with their drivers. Moreover, data show that peer-to-peer support, which may include one-on-one discussions or watching videos on YouTube channels or Facebook groups, is effective at improving dialogue around mental health issues [81]. With technology rapidly advancing, the use of modern communication must be leveraged to reduce the stigma surrounding mental health issues as well as to improve access to care. Future studies are needed to evaluate how effective these strategies are in reducing feelings of isolation and depression. Regarding public policy aimed at improving work conditions, specific focus areas should be work organization features, such as scheduling practices and payment structures, which can be improved through improved recruitment of new LTHD. With more LTHD, this could conceivably reduce the number of hours worked and ultimately improve their mental health.

### 4.2. Prong 2: Future Avenues of Research and Prevention within Novel Research Frameworks

Lessons learned during the COVID-19 pandemic, in conjunction with persistent and worsening mental health outcomes and disparities among specific occupations, provide further impetus for novel approaches to research and prevention. Generally, novel approaches could potentially take any number of forms, ranging from epistemic and pragmatic shifts to a broader paradigmatic shift.

First, abundant opportunities exist to improve worker mental health and well-being by extending beyond the typical domains of current research and drawing insights from other disciplines. For example, syndemic theory, which has roots in the public and population health fields, could be applied to better understand the emergence and persistence of disparate mental health outcomes across occupations. Syndemics are defined as the clustering, due to contextual and social factors, of multiple and adversely interacting disease states within populations [73]. The co-occurrence of these interacting diseases leads to increased burden and vulnerability in these populations that is then exacerbated by those same contextual and social factors that first led to the emergence of the disease cluster; in turn, resulting disease outcomes impact those broader factors and create a reinforcing loop that perpetuates disparities [73]. When applied to safety outcomes, a syndemic perspective can: (1) conceptualize the emergence of these interacting adverse safety and health states as the product of dynamic structural policies and forces that have unfolded over time; (2) anticipate how these adverse states may influence population vulnerabilities and shape injury and disease disparities in a non-linear manner; and (3) identify innovative and holistic multi-level prevention efforts [73]. The presence of latent syndemics among LHTD, including stress-related syndemics [13], has previously been pondered [82].

Similarly, approaches that are typically in the domain of safety science could plausibly be extended to address the work organization–stress–mental health nexus. For example, elements of drift-into-failure—where minute changes among actors in complex systems lead to gradual deterioration of system adaptive capacity and resilience [83]—could be extended to understand how gradual changes in work organization shape exposures to job stress and lead to ‘failures’ that manifest as mental health disorders. Resilience engineering, another complexity-grounded approach to safety that emphasizes successful adaptive responses to novel safety events [84], could similarly be applied to mitigate mental health risks by designing organizations and workplaces that engender psychological resilience among workers. Additionally, the field of cybernetics, with its emphasis on circular processes and circular causality [85], may shed light on how to better align workers with key work organization characteristics, particularly in regard to key feedback mechanisms that may be present that drive the emergence of job stress and mental health disorders. This list is certainly not inclusive, but rather reflects a few of the novel pathways that could potentially be explored.

Finally, a broader paradigm shift may provide numerous novel avenues for basic and applied research into the nexus of work organization, occupational stress, and mental health and well-being. Currently, the majority of studies are guided by research frameworks that seek to identify ‘risk factors’ [73,86] by assuming that such phenomena are best understood by reducing them into component parts—i.e., reductionism [87]. Those parts are then examined in isolation using study designs that emphasize variable isolation (e.g., randomized experiments) [88], and the cumulative understanding that emerges from these studies is then aggregated to provide an understanding of the ‘whole’ [89,90]. The very broad line of research that has emerged from this paradigm has generated numerous meaningful findings. However, the dominance of this paradigm may limit insights into the causality of mental health and well-being outcomes and, in turn, diminish returns from well-intended preventive interventions. For example, because these approaches are predicated on the assumption of linearity—that cause–effect relationships among those isolated parts exhibit proportionality [89]—this paradigm is not well-equipped to capture cyclical connections, such as those found between mental health and physical health [91].

As a novel paradigm, complex systems approaches constitute a novel set of theoretical, methodological, and analytical approaches that may lead to novel insights into how poor mental health and well-being outcomes emerge from work organization and occupational stress, which can lead to high-leverage preventive solutions. In particular, complex systems perspectives can transcend the limitations of prevailing approaches by emphasizing bidirectional interactions and interdependencies between system components [90]. Using methodologies that are endemic to complex systems approaches—namely, dynamic simulation modeling—basic and applied counterfactuals can be tested to examine complexity-grounded theories and preventive solutions [92]. Complex systems approaches have been successfully used in other occupational health and safety contexts [93], including LHTD safety [94], which suggests their potential for worker mental health and well-being as well.

## 5. Conclusions

The nexus of work organization, occupational stress, and mental health is a critical consideration for occupational health and safety. However, significant gaps in understanding necessitate concerted efforts to ensure that prevention strategies can meet known and emerging challenges to worker mental health and well-being in the 21st century. The contributions on behalf of the authors who have submitted manuscripts to this Special Issue constitute meaningful steps to bridging gaps in understanding and informing efficacious preventive solutions to improve mental health and well-being across a broad array of workers across the world. It is anticipated that this commentary, and this Special Issue more broadly, will both echo numerous other calls for building knowledge and engaging in this area and motivate further research within complementary current and novel research frameworks.

## Data Availability

Not applicable.
